# Comparative performance of Gemini and DeepSeek large language models in surgical decision support for mastoidectomy in chronic otitis media with cholesteatoma

**DOI:** 10.3389/fneur.2026.1959170

**Published:** 2026-10-07

**Authors:** Hüseyin Işık, Deniz Baklacı, Gökhan Furkan Kılıç, Seda Nur Saka, Duygu Erdem

**Affiliations:** Department of Otolaryngology, Faculty of Medicine, Zonguldak Bülent Ecevit University, Zonguldak, Türkiye

**Keywords:** artificial intelligence, cholesteatoma, clinical validation, DeepSeek, Gemini, large language models, mastoidectomy, surgical decision support

## Abstract

**Objective:**

To compare the clinical accuracy of two state-of-the-art Large Language Models (LLMs), Gemini Pro v3.1 and DeepSeek v3.2, in providing surgical decision support and postoperative management for mastoidectomy using expert-validated real patient data.

**Methods:**

A retrospective analysis included 121 mastoidectomy cases performed for chronic otitis media with cholesteatoma. Clinical case scenarios, including otoscopy, pure-tone audiometry, and radiological findings, were presented to both models. AI-generated responses were evaluated using a 100-point scoring system developed by four otolaryngologists in two domains: surgical planning and postoperative management. Paired-sample t-tests compared model performance, while Mann–Whitney *U* and Kruskal-Wallis tests assessed the effects of clinical variables.

**Results:**

No significant difference was found between Gemini and DeepSeek in surgical planning (76.07% vs. 73.97%, *p* = 0.258). However, Gemini significantly outperformed DeepSeek in postoperative management (67.85% vs. 62.11%, *p* = 0.001). Both models achieved significantly higher scores in surgical planning than postoperative care. DeepSeek demonstrated significantly higher surgical accuracy in patients with comorbidities (*p* = 0.004) and postoperative complications (*p* < 0.001). Surgical approach significantly influenced DeepSeek’s performance (*p* = 0.001), with better results in microscopic than endoscopic cases.

**Conclusion:**

Both LLMs performed well in surgical planning but were less effective in postoperative management. Gemini demonstrated superior performance in postoperative recommendations, whereas DeepSeek benefited from clearer indicators of disease severity. These findings support the potential of LLMs as adjunctive tools in otologic surgical decision-making while emphasizing the need for expert clinical oversight.

## Introduction

The historical progression of ear surgery has demonstrated a profound transformation, moving from the rudimentary interventions of the past to the highly sophisticated microscopic and endoscopic procedures utilized today (1, 2). Mastoidectomy remains a fundamental pillar in the management of atticoantral chronic otitis media with cholesteatoma, with its primary objectives being the total eradication of disease, the prevention of life-threatening intracranial complications, and the establishment of a safe, dry ear environment (3, 4).

Despite these well-established clinical goals, the strategic choice between Canal Wall Up (CWU) and Canal Wall Down (CWD) techniques continues to be governed by specific clinical indicators, such as the extent of the cholesteatoma and the structural integrity of the posterior ear canal wall (5, 6).

Clinical complexity is notably intensified in scenarios requiring revision mastoidectomy (7, 8). Often necessitated by residual or recurrent disease, revision procedures are characterized by distorted anatomical landmarks, extensive scar tissue formation, and an inherently higher risk of intraoperative complications (9, 10). Furthermore, the postoperative phase demands a rigorous management strategy, incorporating specialized modalities such as non-echo-planar diffusion-weighted magnetic resonance imaging and structured protocols to monitor for expected findings or late-stage sequelae (11, 12). In this context, strict adherence to evidence-based clinical guidelines and continuous performance assessment are indispensable for achieving optimal anatomic and functional outcomes (13, 14).

Parallel to these clinical advancements, the landscape of surgical decision-making is being fundamentally reshaped by the integration of Artificial Intelligence (AI) and Large Language Models (LLMs). The application of AI in craniofacial and oral-maxillofacial surgery has already demonstrated significant potential, extending beyond technical aids like 3D printing to encompass complex preoperative planning and postoperative counseling (15, 16). Comparative evaluations across various LLM architectures have suggested that these models can be highly effective in answering perioperative care questions and translating complex medical information into actionable insights (17–19). Within the specific domain of otolaryngology, head and neck surgery, the diagnostic fidelity and consistency of models such as ChatGPT, Gemini, and DeepSeek with established pediatric and adult guidelines have become a focus of academic scrutiny (20, 21).

Recent systematic reviews and surveyed data indicate that while LLMs show promise in synthesizing medical knowledge, their performance is highly contingent upon the clinical specialty and the level of reasoning required for the specific task (22, 23). For instance, benchmarks involving models like DeepSeek-R1 and GPT-4o in otolaryngology and thyroid surgery have highlighted their potential role as adjunct clinical decision support tools (24–26). However, critical limitations regarding the quality of postoperative instructions and the ability to address the personalized rehabilitation needs of head and neck surgery patients remain significant challenges that require further investigation (27, 28).

Despite the expanding literature on AI in otolaryngology, there is a distinct lack of comparative data regarding the performance of different LLM architectures in the highly structured and technically demanding context of mastoidectomy. This study was designed to evaluate and compare the predictive accuracy of Gemini and DeepSeek models across 121 expert-validated clinical cases. By assessing performance in surgical procedure prediction and postoperative management protocols, while accounting for clinical variables such as comorbidity status, surgical approach, and the presence of complications, this investigation seeks to delineate the current boundaries and reliability of AI reasoning in otologic surgery.

## Method

### Ethical framework and study oversight

This study was conducted following the ethical standards outlined in the Declaration of Helsinki. The research protocol was formally reviewed and approved by the Local Institutional Ethics Committee (Approval No.:2025/19). To protect patient privacy, all clinical records, audiometric data, and radiological images were fully anonymized prior to their integration into the artificial intelligence processing pipeline.

### Clinical cohort and data acquisition

Patients who underwent mastoidectomy for chronic otitis media with cholesteatoma between January 2020 and December 2025 at the Department of Otolaryngology, Zonguldak Bülent Ecevit University Hospital, were retrospectively identified. Consecutive patients with complete medical records and regular postoperative follow-up were eligible for inclusion. Patients who underwent tympanoplasty without mastoidectomy, those with incomplete clinical documentation, and those who failed to attend regular postoperative follow-up visits were excluded. A total of 121 eligible patients met the study criteria and were included in the final analysis.

For each patient, a comprehensive clinical vignette was constructed from hospital records. These profiles included detailed patient histories, otoscopic findings, and complete preoperative diagnostic workups. Where available, non-EPI diffusion-weighted magnetic resonance imaging findings were also included to define the extent of the cholesteatoma.

### AI implementation and prompting strategy

Two frontier LLMs (Gemini Pro v3.1. and DeepSeek v3.2.) were utilized as the experimental subjects. Both are general-purpose frontier large language models that acquire their medical knowledge during large-scale pre-training without domain-specific fine-tuning or retrieval-augmented generation. Both were accessed through their standard web interfaces between June and July 2026. Each of the 121 clinical cases was presented to the models via a standardized prompt in an independent query session with zero-shot prompting across two structured phases mirroring the scoring rubric:

1 Surgical Plan: The necessity of mastoidectomy vs. limited atticotomy/atticoantrotomy, CWU vs. CWD selection, operative technique (inside-out vs. outside-in), modality (endoscopic vs. microscopic), ossicular reconstruction, mastoid obliteration, and cul-de-sac closure.
2 Postoperative Plan: Timing of audiometry, indication and selection of follow-up imaging (modality and month), packing removal timing, and prophylactic antibiotic regimen and duration.

### Expert validation and scoring system

To evaluate the clinical reliability of the AI-generated responses, all outputs were independently assessed by four board-certified otolaryngologists, each with more than 10 years of experience in otologic surgery. The evaluators were blinded to the identity of the language models during the assessment process. Prior to model querying, the four surgeons established the reference standard for each case by reviewing the anonymized preoperative records (history, otoscopy, pure-tone audiometry, temporal-bone CT, and non-EPI DWI-MRI where available) and reaching a collective consensus on the surgical and postoperative management strategy. This consensus decision represented the actual procedure performed. A structured scoring rubric (Table 1), developed by expert consensus according to current evidence-based recommendations and routine clinical practice, was used to compare each AI-generated response with the reference management strategy. Point weights were assigned in proportion to clinical impact on complete disease eradication, patient safety, and recurrence prevention across two independent 100-point domains:

- Surgical Accuracy Domain (100 pts): Evaluated eight critical decisions: mastoidectomy necessity (15 pts), limited atticotomy/atticoantrotomy alternative (15 pts), CWU vs. CWD selection (30 pts), technique (inside-out vs. outside-in, 10 pts), modality (endoscopic vs. microscopic, 10 pts), hearing/ossicular reconstruction (10 pts), mastoid obliteration (5 pts), and cul-de-sac closure (5 pts).
- Postoperative Management Domain (100 pts): Evaluated six categories: timing of postoperative audiometry (20 pts., repeat testing at 8–12 weeks after complete packing resorption and canal healing); follow-up imaging indication (20 pts) and modality/timing (5 pts) tailored to the surgical technique (routine non-EPI DWI-MRI at ~6 months for closed CWU/obliterated cavities vs. direct otomicroscopic surveillance without routine MRI for open CWD cavities); packing removal timing (20 pts); and the regimen (20 pts) and duration (15 pts) of prophylactic antibiotics.

Any response that contradicted surgical safety, risked residual disease, omitted indicated imaging, or proposed inappropriate modalities resulted in point deductions.

**Table 1:** Expert-consensus scoring rubric.

**Decision/item**	**Category**	**Points**
Surgical accuracy domain		
Perform a mastoidectomy?	Indication	15
Limited atticotomy/atticoantrotomy instead of mastoidectomy?	Extent	15
Canal wall up vs. canal wall down?	Canal-wall status	30
Inside-out vs. outside-in?	Technique	10
Endoscopic vs. microscopic?	Modality	10
Hearing / ossicular reconstruction?	Reconstruction	10
Mastoid obliteration?	Obliteration	5
Cul-de-sac (blind-sac) closure?	Closure	5
Surgical domain total		100
Postoperative management domain		
When to obtain audiometry?	Audiometry timing	20
Request follow-up imaging?	Imaging need	20
Which imaging modality and at which month?	Imaging type/timing	5
When to remove the packing?	Packing removal	20
Topical and oral prophylactic antibiotics?	Antibiotic use	20
Duration of prophylactic antibiotics?	Antibiotic duration	15
Postoperative domain total		100

### Statistical analysis

Data were analyzed using IBM SPSS Statistics. Domain accuracy scores were normally distributed and compared using paired-samples t-tests for inter-model and intra-model comparisons. Non-parametric tests (Mann–Whitney U and Kruskal-Wallis) were employed for subgroup comparisons across clinical variables. Correlations between domains were evaluated using Pearson’s correlation coefficient (r). A *p*-value of <0.05 was considered statistically significant.

## Results

### Patient demographics and clinical profile

The study cohort comprised 121 patients who underwent mastoidectomy, with a mean age of 45.0 ± 14.8 years. A descriptive analysis of the demographic data indicated a relatively balanced gender distribution, though males represented a slight majority (55.4%). The cohort was composed overwhelmingly of acquired cholesteatoma (*n* = 117, 96.7%), with congenital cases accounting for 3.3% (*n* = 4). Clinical history revealed that nearly half of the participants (48.8%) were active smokers, and systemic comorbidities were present in 32.2% (*n* = 39, including hypertension, diabetes mellitus, coronary artery disease, COPD, and pulmonary thromboembolism). Disease-related or postoperative complications occurred in 18.2% (*n* = 22, including facial nerve paralysis, labyrinthine fistula, and one advanced case with Bezold/Citelli abscesses and sigmoid sinus thrombophlebitis). Regarding surgical characteristics, Canal Wall Down Tympanomastoidectomy (CWDTM) was the most frequently performed procedure (59.5%), followed by atticotomy/atticoantrotomy (18.2%). The surgical approach was predominantly microscopic (92.6%), and a notable 18.2% of the cohort required revision surgery due to complications or recurrence.

### Comparative performance of LLM architectures

The core of our analysis focused on comparing the evaluative accuracy of the Gemini and DeepSeek LLMs against expert clinical validation. The scoring metrics for both surgical and postoperative dimensions are summarized in Table 2.

**Table 2:** Statistical comparison of accuracy scores between Gemini and DeepSeek (*n* = 121).

**Performance domain**	**Model**	**Mean ± SD (%)**	**Median**	**95% CI**	***t*-value**	***p*-value**
Surgical accuracy	DeepSeek	73.97 ± 21.00	80	[70.18, 77.76]	−1.137	0.258
	Gemini	76.07 ± 17.84	80	[72.85, 79.28]		
Postoperative accuracy	DeepSeek	62.11 ± 16.79	55	[59.08, 65.14]	−3.390	**0.001**
	Gemini	67.85 ± 18.32	75	[64.55, 71.15]		

Bold values indicate statistically significant results (*p* < 0.05).

In terms of surgical procedural accuracy, Gemini achieved a numerically higher mean score (76.07 ± 17.84) compared to DeepSeek (73.97 ± 21.00). However, a paired-samples t-test demonstrated that this disparity was not statistically significant [*t*(120) = −1.137, *p* = 0.258]. The 95% confidence interval for the mean difference ranged from −5.78 to 1.56, encompassing zero, which further suggests that both models provide comparable reliability in predicting intraoperative surgical steps.

Conversely, a sharp divergence in performance was observed within the postoperative treatment domain. Gemini demonstrated a significantly higher level of accuracy (67.85 ± 18.32) relative to DeepSeek (62.11 ± 16.79). This mean difference of 5.74 percentage points was statistically robust [*t*(120) = −3.390, *p* = 0.001] with a 95% confidence interval of −9.10 to −2.39. These results indicate that while both models navigate surgical techniques with similar proficiency, Gemini maintains a superior grasp of postoperative management and clinical follow-up protocols.

### Inter-model correlation analysis

Correlation assessments were conducted to determine the degree of synchrony between the two models across identical patient cases. A moderate and statistically significant positive correlation was identified for both surgical accuracy (*r* = 0.458, *p* < 0.001) and postoperative predictions (*r* = 0.439, *p* < 0.001). This suggests that the models do not operate in complete isolation; rather, they exhibit a shared sensitivity to case complexity, tendentially yielding similar performance fluctuations when faced with high-risk or atypical patient profiles.

### Intra-model performance divergence

A secondary analysis was conducted to evaluate how each specific model performed across different clinical phases. Intriguingly, both Gemini and DeepSeek exhibited a consistent performance drop when transitioning from surgical procedural predictions to postoperative management strategies. For the DeepSeek architecture, the mean surgical accuracy (73.97 ± 21.00%) was substantially higher than its postoperative accuracy (62.11 ± 16.79%). A paired-samples t-test confirmed that this internal discrepancy was statistically significant [*t*(120) = 4.329, *p* < 0.001], with a mean difference of 11.86 percentage points (95% CI: 6.44–17.28). A similar, albeit slightly less pronounced, trend was observed in the Gemini model. Gemini’s surgical accuracy (76.07 ± 17.84%) also outweighed its postoperative performance (67.85 ± 18.32%), yielding a statistically significant difference [*t*(120) = 3.044, *p* = 0.003] and a mean gap of 8.22 points (95% CI: 2.88–13.57). These findings, detailed in Table 3, suggest that while LLMs are relatively proficient in articulating structured surgical steps, they struggle with the inherent heterogeneity and clinical fluidity of postoperative care.

**Table 3:** Within-model comparison of surgical and postoperative accuracy scores.

**Model**	**Domain comparison**	**Mean ± SD (%)**	**Mean difference**	***t*-value**	***p*-value**
DeepSeek	Surgery vs. Post-op	73.97 ± 21.00 vs. 62.11 ± 16.79	11.86	4.329	**<0.001**
Gemini	Surgery vs. Post-op	76.07 ± 17.84 vs. 67.85 ± 18.32	8.22	3.044	**0.003**

Bold values indicate statistically significant results (*p* < 0.05).

### Correlation disparity and clinical complexity

To further investigate the relationship between these two domains, we calculated the intra-model correlations between surgical and postoperative accuracy. As shown in Table 4, a modest but significant negative correlation was identified for both models (DeepSeek: *r* = −0.263; Gemini: *r* = −0.350). This inverse relationship suggests a critical clinical finding: cases where the models accurately predicted surgical maneuvers were not necessarily matched by high-fidelity postoperative recommendations. In fact, as surgical accuracy improved in specific cases, there was a measurable tendency for postoperative accuracy to diminish.

**Table 4:** Intra-model correlation between surgical and postoperative performance.

**Model performance relationship**	**Pearson correlation (r)**	***p*-value**
DeepSeek (Surgical vs. Post-op)	−0.263	**0.004**
Gemini (Surgical vs. Post-op)	−0.350	**<0.001**

Bold values indicate statistically significant results (*p* < 0.05).

### Impact of surgical complexity

To further highlight the robustness of LLM performance, accuracy scores were categorized according to revision surgery history. Given the inherent complexities associated with revision mastoidectomy, such as distorted anatomical reference points, scar tissue planes, and heterogeneous clinical presentations, the impact of these factors on the predictive accuracy of the models was investigated. As detailed in Table 5, a Mann–Whitney *U* analysis revealed no statistically significant disparity in surgical accuracy scores between primary and revision cases for either DeepSeek (*p* = 0.505) or Gemini (*p* = 0.583). While statistical significance was not achieved for postoperative accuracy, a notable directional trend emerged (DeepSeek *p* = 0.073; Gemini *p* = 0.108).

**Table 5:** Comparison of AI accuracy scores stratified by revision surgery status.

**Outcome metric**	**Revision status**	**Mean rank**	***U***	***Z***	***p*-value**
DeepSeek surgical accuracy	No (*n* = 99)	60.01	990.5	−0.666	0.505
	Yes (*n* = 22)	65.48			
Gemini surgical accuracy	No (*n* = 99)	60.18	1008.0	−0.549	0.583
	Yes (*n* = 22)	64.68			
DeepSeek postoperative accuracy	No (*n* = 99)	63.52	840.0	−1.791	0.073
	Yes (*n* = 22)	49.68			
Gemini postoperative accuracy	No (*n* = 99)	63.33	858.0	−1.609	0.108
	Yes (*n* = 22)	50.50			

### Predictive performance in complex clinical scenarios

A critical dimension of this study was to determine whether the occurrence of postoperative complications influenced the predictive accuracy of the AI models. Data regarding the impact of complications on model performance is synthesized in Table 6. The most compelling finding emerged from the analysis of DeepSeek’s surgical accuracy. A Mann–Whitney *U* test revealed a statistically significant difference (*U* = 563.5, Z = −3.555, *p* < 0.001), where cases that eventually encountered complications were associated with substantially higher mean ranks (84.89) compared to those without complications (55.69). Similar trends were observed in Gemini’s surgical accuracy (*p* = 0.063) and DeepSeek’s postoperative recommendations (*p* = 0.084).

**Table 6:** AI accuracy scores stratified by postoperative complication status.

**Performance metric**	**Complication status**	**Mean rank**	***U***	***Z***	***p*-value**
DeepSeek surgical accuracy	No (*n* = 99)	55.69	563.5	−3.555	**<0.001**
	Yes (*n* = 22)	84.89			
Gemini surgical accuracy	No (*n* = 99)	58.22	814.0	−1.862	0.063
	Yes (*n* = 22)	73.50			
DeepSeek postoperative accuracy	No (*n* = 99)	58.58	849.0	−1.727	0.084
	Yes (*n* = 22)	71.91			
Gemini postoperative accuracy	No (*n* = 99)	59.95	985.5	−0.721	0.471
	Yes (*n* = 22)	65.70			

Bold values indicate statistically significant results (*p* < 0.05).

### The role of systemic comorbidities in predictive accuracy

To explore the potential influence of the patients’ general health status on algorithmic performance, we stratified the accuracy scores according to the presence of systemic comorbidities (*n* = 39, 32.2%). The comparative analysis, summarized in Table 7, revealed a significant correlation between comorbidity status and DeepSeek’s surgical predictive fidelity (mean rank 74.38 vs. 54.63; *U* = 1077.0, Z = −2.914, *p* = 0.004). In contrast, Gemini did not exhibit statistically significant variation (*p* = 0.146), nor did postoperative domains (DeepSeek *p* = 0.749; Gemini *p* = 0.216).

**Table 7:** Influence of comorbidity status on AI model accuracy.

**Performance Metric**	**Comorbidity**	**Mean rank**	***U***	***Z***	***p*-value**
DeepSeek surgical accuracy	No (*n* = 82)	54.63	1077.0	−2.914	**0.004**
	Yes (*n* = 39)	74.38			
Gemini surgical accuracy	No (*n* = 82)	57.83	1339.0	−1.423	0.146
	Yes (*n* = 39)	67.67			
DeepSeek postoperative accuracy	No (*n* = 82)	61.66	1545.0	−0.321	0.749
	Yes (*n* = 39)	59.62			
Gemini postoperative accuracy	No (*n* = 82)	58.37	1383.5	−1.238	0.216
	Yes (*n* = 39)	66.53			

Bold values indicate statistically significant results (*p* < 0.05).

### Impact of surgical modality on algorithmic precision

To assess the influence of the operative technique, we categorized cases into microscopic, endoscopic, and combined approaches. As shown in Table 8, a Kruskal-Wallis analysis revealed a statistically significant variation in DeepSeek’s surgical accuracy based on the approach (*H* = 14.650, df = 2, *p* = 0.001). Specifically, DeepSeek performed optimally in cases utilizing a microscopic approach (Mean Rank: 64.37) but demonstrated a marked decline in accuracy for endoscopic procedures (Mean Rank: 15.94). In contrast, Gemini’s surgical accuracy did not show a statistically significant dependence on the surgical approach (*p* = 0.088). Postoperative accuracy for both models remained relatively stable across modalities (DeepSeek *p* = 0.096; Gemini *p* = 0.351).

**Table 8:** Comparison of AI accuracy scores according to surgical approach.

**Outcome metric**	**Microscopic (*n* = 112)**	**Endoscopic (*n* = 8)**	**Combined (*n* = 1)**	***H***	***p*-value**
DeepSeek surgery accuracy	64.37	15.94	44.50	14.650	**0.001**
Gemini surgery accuracy	62.69	35.25	77.50	4.868	0.088
DeepSeek post-op accuracy	60.35	76.88	50.50	4.689	0.096
Gemini post-op accuracy	60.21	75.38	35.00	2.094	0.351

Bold values indicate statistically significant results (*p* < 0.05).

### Final synthesis of model comparisons

The cumulative data indicate a distinct performance hierarchy between the two architectures. When directly comparing the models, Gemini demonstrated significantly higher accuracy in postoperative treatment recommendations than DeepSeek (*p* = 0.001). However, no significant difference was observed between the two models in the domain of surgical procedure prediction (*p* = 0.258). A summary of the primary performance metrics is provided in Table 9. Both models consistently achieved higher scores in predicting surgical steps compared to postoperative management recommendations (DeepSeek *p* < 0.001; Gemini *p* = 0.003).

**Table 9:** Summary comparison of mean accuracy scores between models.

**Model output domain**	**DeepSeek Mean ± SD**	**Gemini Mean ± SD**	***p*-value**
Surgical procedure accuracy	73.97 ± 21.00%	76.07 ± 17.84%	0.258
Postoperative care accuracy	62.11 ± 16.79%	67.85 ± 18.32%	**0.001**

Bold values indicate statistically significant results (*p* < 0.05).

## Discussion

The integration of LLMs into surgical decision-making represents a pivotal shift in otolaryngological practice, yet the nuances of their clinical reliability remain under rigorous debate. The findings of this study demonstrate that both Gemini and DeepSeek exhibit substantial proficiency in articulating structured surgical procedures for mastoidectomy, achieving comparable accuracy levels. This alignment suggests that LLMs have successfully internalized the core surgical principles established in foundational texts and clinical guidelines, such as those regarding the necessity of mastoidectomy in the presence of extensive cholesteatoma (3, 4, 14). However, a significant performance gap was observed in the domain of postoperative management, where Gemini showed a distinct superiority over DeepSeek. This disparity may be attributed to Gemini’s advanced synthesis capabilities in handling longitudinal care protocols, which involve a complex interplay of temporal variables and medication regimens that often diverge from the more rigid, step-by-step logic of intraoperative planning (22, 23, 28).

A particularly striking finding of this investigation is the paradoxical improvement in DeepSeek’s surgical accuracy when confronted with clinical complexity. In cases characterized by systemic comorbidities or postoperative complications, DeepSeek’s predictive fidelity increased significantly. This phenomenon suggests that certain AI architectures may be highly sensitive to explicit clinical signals, clear indicators of severity that narrow the range of appropriate surgical responses (24, 26). In contrast, the significant variation in DeepSeek’s performance based on the surgical approach, with a marked preference for microscopic over endoscopic cases, highlights a potential limitation in its training data or reasoning pathways regarding evolving surgical modalities (13, 20).

The comparative diagnostic and management capabilities observed in this study align with recent literature highlighting the potential of advanced reasoning models like DeepSeek-R1 and Gemini in specialized fields (12, 16). Nevertheless, the persistent difficulty both models faced in postoperative care, scoring significantly lower than in surgical planning, underscores the inherent difficulty of AI in navigating the gray areas of clinical follow-up. While surgical steps for cholesteatoma are often governed by anatomical erodibility and disease extent, postoperative management requires a more heuristic approach that accounts for wound healing variability and patient-specific risks (10, 12).

The strength of this analysis lies in its use of expert-validated, real-world clinical vignettes, which move beyond theoretical questions to test the models against the practical realities of otologic surgery. The ability of LLMs to predict correct interventions in nearly 75% of surgical scenarios suggests they are rapidly approaching a level of utility that could support residents and junior surgeons in preoperative planning (24, 27). However, the divergence between intraoperative technical accuracy and postoperative managerial accuracy serves as a critical benchmark for the current state of AI in otolaryngology, suggesting that while the models are adept at performing a virtual surgery, the longitudinal stewardship of the surgical patient remains a predominantly human domain.

## Conclusion

This study provides a comprehensive benchmark for the utility of large language models in the specialized domain of otologic surgery, revealing that both Gemini and DeepSeek possess robust capabilities in predicting structured surgical procedures. The most significant takeaway is the consistent proficiency of these models in articulating intraoperative plans, contrasted with a measurable decline in accuracy regarding longitudinal postoperative management. Furthermore, the observation that clinical complexity acts as a performance enhancer for certain architectures, such as DeepSeek, suggests that AI reasoning may be inherently tied to the explicitness of clinical severity signals.

Despite these promising findings, several limitations must be acknowledged to contextualize the results. First, the retrospective nature of the case vignettes and the single-center data source may limit the generalizability of the findings across different surgical cultures or healthcare systems. Additionally, while the scoring rubric was expert-validated, the inherent subjectivity of surgical gold standards remains a factor, as operative decisions in complex mastoidectomy cases can occasionally vary even among senior clinicians. The relatively small sample size in specific subgroups, such as the endoscopic and combined surgical approaches, also necessitates caution when interpreting the impact of operative modality on model accuracy.

Looking forward, these results underscore the need for more nuanced and multi-center prospective studies to further delineate the boundaries of AI-assisted surgical decision support. Future research should focus on the integration of multimodal data, incorporating raw radiological images and intraoperative video feeds, rather than relying solely on text-based clinical vignettes. Ultimately, while this study positions LLMs as powerful adjuncts for surgical planning and resident education, it reaffirms that the multifaceted stewardship of the postoperative patient remains an area where expert human judgment is currently irreplaceable.

Another limitation is that although all AI-generated responses were independently reviewed by four experienced otologists, formal inter-rater reliability analysis was not performed. Future studies incorporating validated assessment instruments together with inter-rater agreement statistics would further strengthen the methodological robustness of AI evaluation in surgical decision-support research.

## Data Availability

The original contributions presented in the study are included in the article/supplementary material, further inquiries can be directed to the corresponding author/s.

## References

[1] Bento RF de Oliveira Fonseca AC. A brief history of mastoidectomy. Int Arch Otorhinolaryngol. (2013) 17:168–78. doi: 10.7162/S1809-97772013000200009 PMC4423283 25992009

[2] Nadol JB. Revision mastoidectomy. Otolaryngol Clin N Am. (2006) 39:723–40. doi: 10.1016/j.otc.2006.05.003, 16895781

[3] Bennett M Warren F Haynes D. Indications and technique in mastoidectomy. Otolaryngol Clin N Am. (2006) 39:1095–113. doi: 10.1016/j.otc.2006.08.012 17097435

[4] Fisch U May JS Linder T. Tympanoplasty, Mastoidectomy, and Stapes Surgery. 2nd ed. Stuttgart, Germany: Thieme (2010).

[5] Berçin S Kutluhan A Bozdemir K Yalçiner G Sari N Karameşe Ö. Results of revision mastoidectomy. Acta Otolaryngol. (2009) 129:138–41. doi: 10.1080/00016480802140893 18607914

[6] Sonnenschein R. The development of mastoidectomy. Ann Med Hist. (1936) 8:500. PMC7939872 33943521

[7] Migirov L Eyal A Kronenberg J. Intracranial complications following mastoidectomy. Pediatr Neurosurg. (2004) 40:226–9. doi: 10.1159/000082296 15689642

[8] Kos MI Montandon P Castrillon R Guyot JP. Anatomic and functional long-term results of canal wall-down mastoidectomy. Ann Otol Rhinol Laryngol. (2004) 113:872–6. doi: 10.1177/000348940411301105 15562896

[9] Sethia R Kerwin TF Wiet GJ. Performance assessment for mastoidectomy: state of the art review. Otolaryngol Head Neck Surg. (2017) 156:61–9. doi: 10.1177/0194599816670886, 27677598 PMC5209289

[10] Aristizabal J Puac P Zamora C Castillo M. Expected findings and complications after tympanoplasty and mastoidectomy. Neurographics. (2019) 9:220–30. doi: 10.3174/ng.1800062

[11] Grillo R Llanos AH Costa C Melhem-Elias F. Large language models comparison in oral and maxillofacial surgery. Br J Oral Maxillofac Surg. (2025). 64:43–49. doi: 10.1016/j.bjoms.2025.08.015 41076417

[12] Prasad S Langlie J Pasick L Chen R Franzmann E. Evaluating advanced AI reasoning models: ChatGPT-4.0 and DeepSeek-R1 diagnostic performance in otolaryngology: a comparative analysis. Am J Otolaryngol. (2025) 46:104667. doi: 10.1016/j.amjoto.2025.104667, 40367837

[13] Filali Ansary R Lechien JR. Clinical decision support using large language models in otolaryngology: a systematic review. Eur Arch Otorrinolaringol. (2025) 282:4325–34. doi: 10.1007/s00405-025-09504-8, 40481345

[14] Rossi NA Corona KK Yoshiyasu Y Hajiyev Y Hughes CA Pine HS. Comparative analysis of GPT-4 and Google Gemini's consistency with pediatric otolaryngology guidelines. Int J Pediatr Otorhinolaryngol. (2025) 193:112336. doi: 10.1016/j.ijporl.2025.112336, 40203537

[15] Munkwitz SE Shah H Iglesias NJ Quan H Riveron S Nayak VV . A review of artificial intelligence in craniofacial surgery: clinical applications beyond 3D printing. J Craniofac Surg. (2026) 37:883–96. doi: 10.1097/SCS.0000000000012277 41773851

[16] Zou L Zhang P Jiang YQ Wang XW Yan XJ Wu JZ . ChatGPT-4o, Gemini advanced and DeepSeek R1 in preoperative decision-making for thyroid surgery: a comparative assessment with human surgeons. Front Oncol. (2025) 15:1590230. doi: 10.3389/fonc.2025.1590230, 41211446 PMC12591979

[17] Sandmann S Hegselmann S Fujarski M Bickmann L Wild B Eils R . Benchmark evaluation of DeepSeek large language models in clinical decision-making. Nat Med. (2025) 31:2546–9. doi: 10.1038/s41591-025-03727-2, 40267970 PMC12353792

[18] Alamri AH AlZabin A Magboul N Alrasheed AS Alokby G Alroqi A. Comparative analysis of artificial intelligence platforms in generating post-operative instructions for endoscopic transnasal skull base surgery. Eur Arch Otorrinolaringol. (2025) 283:243–8. doi: 10.1007/s00405-025-09760-8, 41249683

[19] Maia BKF de Morais EF de Santana Santos T Pagotto LEC. Performance of large language models in preoperative and postoperative counselling for aesthetic facial procedures. Br J Oral Maxillofac Surg. (2026). 64:216–222. doi: 10.1016/j.bjoms.2026.01.002 41633924

[20] Pinto MM Araújo EER Rahhal Z Stanbouly D Grillo R. AI-assisted translation in oral and maxillofacial surgery: a comparative evaluation. J Stomatol Oral Maxillofac Surg. (2026) 127:102717. doi: 10.1016/j.jormas.2026.102717, 41539644

[21] Mangal R Kring T Desai A Bustos V Dadi S Kassira W . Artificial intelligence in craniosynostosis surgery: a comparison of large language models in answering perioperative care questions. J Craniofac Surg. (2026) 37:790–4. doi: 10.1097/scs.0000000000012087 41575385

[22] Marcaccini G Seth I Novo J McClure V Sacks B Lim K . Leveraging artificial intelligence for personalized rehabilitation programs for head and neck surgery patients. Technologies (Basel). (2025) 13:142. doi: 10.3390/technologies13040142

[23] Warrier A Singh R Haleem A Zaki H Eloy JA. The comparative diagnostic capability of large language models in otolaryngology. Laryngoscope. (2024) 134:3997–4002. doi: 10.1002/lary.31434 38563415

[24] Ahn J Kang BG Chang M Yoon S. Applications and future perspectives of large language models in otolaryngology-head and neck surgery: a comprehensive survey. Clin Exp Otorhinolaryngol. (2025) 18:283–95. doi: 10.21053/ceo.2025-00121, 40590109 PMC12588054

[25] Merlino DJ Brufau SR Saieed G Van Abel KM Price DL Archibald DJ . Comparative assessment of otolaryngology knowledge among large language models. Laryngoscope. (2025) 135:629–34. doi: 10.1002/lary.31781, 39305216

[26] Bao JW Jawad MN Pavelchek C Powell WJ Oh IY Gupta A . Large language models and otolaryngology: a review. JAMA Otolaryngol Head Neck Surg. (2024). 152:425–432. doi: 10.1001/jamaoto.2025.5335 41642593

[27] Lechien JR Rameau A. Applications of ChatGPT in otolaryngology–head neck surgery: a state of the art review. Otolaryngol Head Neck Surg. (2024) 171:667–77. doi: 10.1002/ohn.807, 38716790

[28] Buhr CR Smith H Huppertz T Bahr-Hamm K Matthias C Cuny C . Assessing unknown potential quality and limitations of different large language models in the field of otorhinolaryngology. Acta Otolaryngol. (2024) 144:237–42. doi: 10.1080/00016489.2024.2352843, 38781053

